# Clinical characteristics of patients with suspected Alzheimer’s disease within a CSF Aß-ratio grey zone

**DOI:** 10.1186/s42466-023-00262-8

**Published:** 2023-08-03

**Authors:** Dariia Yosypyshyn, Domantė Kučikienė, Inez Ramakers, Jörg B. Schulz, for the Alzheimer’s Disease Neuroimaging Initiative, Kathrin Reetz, Ana Sofia Costa

**Affiliations:** 1 Department of Neurology, University Hospital RWTH Aachen, Pauwelsstr. 30, 52074 Aachen, Germany; 2 JARA Institute Molecular Neuroscience and Neuroimaging, RWTH Aachen & Forschungszentrum Jülich, Aachen, Germany; 3 Department of Psychiatry and Neuropsychology, School for Mental Health and Neuroscience, Alzheimer Center Limburg, Maastricht University, Maastricht, The Netherlands

**Keywords:** Alzheimer’s disease, Cognitive impairment, Memory clinic, ATN, Biomarkers, Validation, Aß_42_/Aß_40_ ratio

## Abstract

**Background:**

The AT(N) research framework for Alzheimer's disease (AD) remains unclear on how to best deal with borderline cases. Our aim was to characterise patients with suspected AD with a borderline Aß_1-42_/Aß_1-40_ ratio in cerebrospinal fluid.

**Methods:**

We analysed retrospective data from two cohorts (memory clinic cohort and ADNI) of patients (n = 63) with an Aß_1-42_/Aß_1-40_ ratio within a predefined borderline area—Q_1_ above the validated cut-off value(grey zone). We compared demographic, clinical, neuropsychological and neuroimaging features between grey zone patients and patients with low Aß_1-42_ (normal Aß ratio but pathological Aß_1-42_, n = 42) and patients with AD (pathological Aß, P-Tau, und T-Tau, n = 80).

**Results:**

Patients had mild cognitive impairment or mild dementia and a median age of 72 years. Demographic and general clinical characteristics did not differ between the groups. Patients in the grey zone group were the least impaired in cognition. However, they overlapped with the low Aß_1-42_ group in verbal episodic memory performance, especially in delayed recall and recognition. The grey zone group had less severe medial temporal atrophy, but mild posterior atrophy and mild white matter hyperintensities, similar to the low Aß_1-42_ group.

**Conclusions:**

Patients in the Aß ratio grey zone were less impaired, but showed clinical overlap with patients on the AD continuum. These borderline patients may be at an earlier disease stage. Assuming an increased risk of AD and progressive cognitive decline, careful consideration of clinical follow-up is recommended when using dichotomous approaches to classify Aß status.

**Supplementary Information:**

The online version contains supplementary material available at 10.1186/s42466-023-00262-8.

## Background

The NIA-AA Research Framework AT(N) classification was developed as an unbiased biomarker-based classification system for Alzheimer's disease (AD) with the aim of more accurately defining its nosology [25]. The AT(N) is a dichotomous classification system for the categorisation of multi-domain biomarker findings based on the presence or absence of amyloid pathology (A), tauopathy (T) and neurodegeneration (N). While several efforts are underway to translate the AT(N) classification into clinical practice [2, 14, 18, 39], an open question, given its dichotomous nature, is how best to deal with borderline cases.

Previous work has proposed the use of peri-threshold biomarker zones or grey zones. These are intervals around cut-off reference values within which the ability to discriminate, for example, between cognitive decline in individuals with normal and abnormal biomarker levels may be lost [7, 8, 38]. However, a significant unmet clinical need remains due to uncertainty about how best to implement such an approach in a clinical context. One difficulty is the lack of clear recommendations on how to define a potential borderline zone. One approach, for example, is to use the measurement error associated with a particular biomarker, typically 5–10% above the cut-off. However, measurement errors margins are highly dependent on the laboratory’s experience and assay performance [34], and few studies have assessed their validity in clinical practice.

To diagnose AD, the AT(N) requires the presence of amyloid-ß (Aß) pathology [1, 25], for which three established biomarkers are currently used: cerebrospinal fluid (CSF) amyloid Aß_1-42_, CSF amyloid Aß_1-42_/Aß_1-40_ ratio, or amyloid positron-emission tomography (PET) [31, 33, 36, 42]. Among the CSF biomarkers, the Aß_1-42_/Aß_1-40_ ratio is considered more reliable than the Aß_1-42_ alone [3, 16, 29, 37, 43]. One the one hand, the Aß_1-42_/Aß_1-40_ ratio minimize bias associated with pre-analytical or analytical factors. One the other hand, the ratio appears to be more sensitive and specific for AD pathology [37].

According to the AT(N) classification, individuals with an Aß_1-42_/Aß_1-40_ ratio slightly above the cut-off value would be classified as amyloid-negative (A-) and therefore as having no AD pathology, regardless of possible discrepancies with the clinical and cognitive profile. To avoid false negatives, it may be helpful for clinicians to establish a grey area to improve the reliability of AD diagnosis when using CSF biomarkers.

Our aim was to characterise an amyloid peri-threshold grey zone group of individuals with suspected AD in terms of demographic, clinical, neuropsychological and neuroimaging features using the CSF Aß_1-42_/Aß_1-40_ ratio to determine amyloid status. In particular, we wanted to investigate how patients in this grey zone differ from those with low Aß_1-42_ (A+) and those with a typical AD biomarker profile (A + T + (N +)).

## Materials and methods

We analysed data from two independent cohorts: a memory clinic cohort (Aachen Memory Database) and data from the Alzheimer’s Disease Neuroimaging Initiative (ADNI).

### Memory clinic cohort (Aachen Memory Database)

The memory clinic cohort includes patients who were consecutively referred to the Memory Clinic of the Department of Neurology at the University Hospital RWTH Aachen (Germany) between July 2009 and October 2020 for diagnostic evaluation of suspected cognitive impairment. All included patients underwent diagnostic CSF analyses for neurodegeneration markers, brain magnetic resonance imaging (MRI), including T1-weigthed, T2-weigthed, fluid-attenuated inversion recovery (FLAIR) and T2* sequences, and comprehensive neuropsychological assessment. Exclusion selection criteria were: (1) unavailable CSF biomarkers; (2) patients with CSF preanalytical errors; (3) patients with other diagnoses (e.g., frontotemporal lobar degeneration, Parkinson’s disease, dementia with Lewy bodies, prion disease, major psychiatric disease, epilepsy, encephalopathy).The clinical diagnosis was made using international diagnostic criteria and national diagnostic guidelines [15] and based on all available diagnostic information, including clinical and neuropsychological assessment, CSF biomarker and MRI results. The study was approved by the local ethics committee (EK 018-19) and conducted in accordance with The Code of Ethics of the World Medical Association (Declaration of Helsinki).

CSF samples were collected as part of routine clinical diagnostic work-up. Polypropylene tubes were used to minimize preanalytical errors in the Aß quantification [11, 41]. CSF parameters were obtained from the Neurochemical Laboratory at the University of Göttingen and included Aß_1-42_, Aß_1-40_, Aß_1-42_/Aß_1-40_ ratio × 10, total Tau protein (T-Tau) and phosphorylated tau protein 181p (P-Tau). All CSF biomarkers were measured using commercially available assays (INNOTEST, Fujirebio, Ghent, Belgium and IBL International, Hamburg, Germany). The pathological reference values were previously validated and used as follows: Aß_1-42_ < 450 pg/mL; Aß_1-42_/Aß_1-40_ ratio < 0.5; T-Tau > 450 pg/mL; and P-Tau > 61 pg/mL [24].

We collected clinical and neuropsychological data including demographics (age, sex, years of education), medical history, presence of vascular risk factors, family history of dementia in first-degree relatives, global cognitive status (Mini-Mental State Examination (MMSE) and/or Montreal Cognitive Assessment (MoCA)), clinical disease severity (Clinical Dementia Rating Scale, CDR), self-report of depressive symptoms (Beck Depression Inventory, BDI-II) and comprehensive neuropsychological assessment results (CERAD-NAB battery). Routine MRI scans were independently reviewed by two raters blinded to clinical information, using the following rating scales: bilateral medial temporal atrophy (MTA) score (range 0–4), posterior atrophy score (Koedam score, range 0–3) and white matter hyperintensities score (Fazekas score, range 0–3). All assessment materials and rating methods have been previously described in studies from the same cohort [12, 26].

To define the grey zone group, we included only patients with both a normal Aß_1-42_/Aß_1-40_ ratio > 0.5 and Aß_1-42_ > 450 pg/ml, i.e., non-pathological Aß parameters according to validated laboratory references. Other CSF parameters, such as P-Tau and T-Tau, were not considered because we were interested in their possible modulatory effect in post hoc sensitivity analyses. We defined the peri-threshold zone—the grey zone, based on the quartile distribution of the data. In the absence of guidelines for establishing such peri-threshold zones, we also used the recommendations derived from the Erlangen score [4, 19], which resulted in similar group compositions. We calculated the first quartile (25%) to define the cut-off value for the grey zone. The upper threshold was defined as an Aß_1-42_/Aß_1-40_ ratio of 0.56, so that the grey zone corresponds to values between 0.5 and 0.56. For the AD group, we selected patients with a biomarker profile corresponding to A + T + (N)+, as well as significant MTA atrophy (MTA score ≥ 2) and a typical neuropsychological profile for AD. We also defined a group of patients with low Aß_1-42_ (normal Aß_1-42_/Aß_1-40_ ratio (> 0.5) but low Aß_1-42_ (< 450 pg/ml), independent of P-Tau or T-Tau CSF concentrations). All groups were matched for sex, mean age and years of education.

### ADNI cohort

We included participants from the ADNI database (NCT00106899). The ADNI project, a multicentre longitudinal study, aims to combine clinical, imaging, genetic, and biochemical biomarkers to develop and validate the measures for the early diagnosis of late-onset AD. Detailed inclusion criteria for ADNI are available at www.adni-info.org. ADNI was approved by the institutional review boards of all participating institutions and all participants provided written informed consent.

For this study, we selected patients with available CSF Aß_1-42_ and Aß_1-40_ data and composite Florbetapir standardized uptake value ratios (SUVRs) from Aß-PET measurements [20, 27]. CSF Aß_1-42_, Aß_1-40_, T-Tau, and P-Tau samples were analysed by the University of Pennsylvania ADNI Biomarker Core Laboratory using the Roche Elecsys immunoassay on the cobas e601 fully automated system with the following cut-off values: Aß_1-42_ < 980 pg/mL, T-Tau > 266 pg/mL, P-Tau > 24 pg/mL [5, 20, 22]. As there is no predefined Aß ratio cut-off for ADNI, we calculated the Aß_1-42_/Aß_1-40_ ratio using Aß-PET results as the reference standard [32]. Aß-PET positivity is defined as Florbetapir SUVR greater than 1.1 at baseline [20]. We calculated the cut-off for the Aß_1-42_/Aß_1-40_ ratio based on ROC analyses using the Youden index and classified 209 Aß-PET-negative cognitively normal (CN) participants and 78 Aß-PET-positive mild cognitive impairment (MCI) and AD patients. The optimal cut-off was found to be 0.0522 (AUC = 0.938, *p* < 0.001). We multiplied the cut-off value by 10 to facilitate comparison with the Aß-ratio units from the memory clinic cohort. To define the grey zone for the Aß_1-42_/Aß_1-40_ ratio within ADNI, we also used a quartile function, as described above. The grey zone for the ADNI cohort was set between 0.522 and 0.733. Group composition followed the procedure described above for the memory clinic cohort.

From the neuropsychological data available in ADNI, we selected the tests that best matched the protocol in the memory clinic cohort, namely Category Fluency, Boston Naming Test or Multilingual Naming Test, Rey Auditory Verbal Learning Test and the Trail Making Test (versions A and B). Visual ratings of the brain MRI scans for the ADNI cohort were also performed according to the procedure described above.

### Statistical analysis

Data are presented as frequencies (percentages) or medians (interquartile ranges, IQR). Non-parametric methods were used for group comparisons, given the sample size and the normality distribution of the data, which was assessed using the Kolmogorov–Smirnov test. Chi-square, Mann–Whitney and Kruskal–Wallis tests were used in the main and sensitivity analyses, depending on the group comparison. Pairwise comparisons were corrected for multiple tests using the Bonferroni correction. Effect size (ES) indicates Cohen's d or Cramer's V for the respective group comparison test.

To increase statistical power, we decided to pool the data from both cohorts, as the majority of preliminary analyses showed no relevant differences between the cohorts. Group comparisons for CSF concentrations were also performed separately by cohort, as the cut-off values were different. We calculated measures of association between Aß_1-42_ concentrations and neuropsychological performance, separately for each group, using Spearman's rank correlation coefficient and controlling for outliers.

Statistical analyses were performed with IBM SPSS (version 28) and MedCalc (version 19.3), and visualizations with Python programming language (version 3.9, using: pandas 1.4.2, seaborn 0.11.2, matplotlib 3.5.1). All tests were two-tailed with an alpha of 0.05 set as the significance threshold.

## Results

### Sample characteristics

**Fig. 1:**
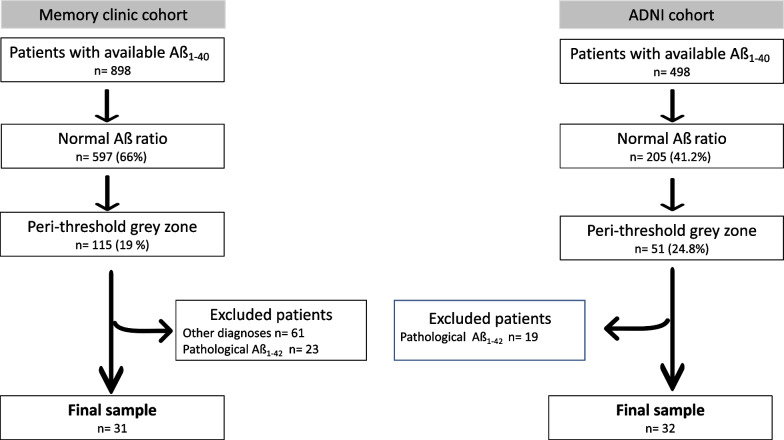
Flow-chart for patient selection for the grey zone group in each cohort (local memory clinic and ADNI)

In both cohorts, 63 patients were identified in the grey zone group, 42 patients in the low Aß_1-42_ group and 80 patients in the AD group (see Fig. 1 for the flowchart of patient selection in the grey zone group).

Patients had a median age at diagnosis of 71.5 years (IQR 68–76) and a median education of 14.5 years (IQR 11–18). As shown in Table 1, there were no differences between the groups with respect to demographic variables and clinical characteristics, including family history, frequency of previous medical conditions, and vascular risk factors.

**Table 1:** Demographic and clinical characterization of patients per group

Variable	Group			Test statistic and *p* value	*ES*
	Grey Zone n = 63	low Aß_1-42_ n = 42	AD n = 80		
*Demographics*					
Age at diagnosis (years), median (IQR)	71 (68–76)	72.4 (67–79)	72 (68–78)	H(2) = 0.450, * p* = 0.799	0.19
Sex (Female), n (%)	40 (63.49%)	19 (45.24%)	41 (51.25%)	X^2^(2) = 3.827,* p* = 0.148	0.14
Education (years), median (IQR)	16 (11–18)	14.5 (12–18)	13 (11–16)	H(2) = 2.519, * p* = 0.284	0.11
History of dementia in first degree relatives, n (%)	21 (33.3%)	21 (50%)	31 (38.75%)	X^2^(2) = 1.481,* p* = 0.477	0.10
*Medical history*, n (%)					
Arterial hypertension	27 (42.9%)	22 (52.4%)	46 (58.2%)	X^2^(2) = 0.876,* p* = 0.645	0.08
Diabetes mellitus	10 (15.9%)	5 (11.9%)	7 (8.8%)	X^2^(2) = 3.025,* p* = 0.220	0.17
Dyslipidaemia	32 (50.8%)	22 (52.4%)	38 (47.5%)	X^2^(2) = 1.054,* p* = 0.591	0.09
OSAS	4 (6.4%)	4 (9.5%)	4 (5.0%)	X^2^(2) = 1.104,* p* = 0.576	0.10
Smoking	8 (12.7%)	6 (14.29%)	13 (16.25%)	X^2^(4) = 1.026,* p* = 0.906	0.10
Depression	13 (20.63%)	6 (14.28%)	18 (22.5%)	X^2^(2) = 0.646,* p* = 0.724	0.07
Ischemic stroke/TIA	2 (3.17%)	5 (11.9%)	6 (7.5%)	X^2^(4) = 3.307,* p* = 0.508	0.13
Coronary heart disease	4 (6.35%)	4 (9.52%)	6 (7.5%)	X^2^(2) = 0.359,* p* = 0.836	0.06
*Clinical severity*, n (%)				X^2^(8) = 46.459,* p* < 0.001	0.38
CDR total score 0	28 (44.4%)	14 (33.3%)	0 (0%)		
CDR total score 0.5	17 (26.98%)	11 (26.19%)	20 (25%)		
CDR total score ≥ 1	18 (28.57%)	17 (40.48%)	60 (75%)		

AD, Alzheimer’s disease, CDR, Clinical dementia rating scale, ES, effect sizes, IQR, Interquartile range, OSAS, obstructive sleep apnoea syndrome, TIA, transitory ischemic attack

Overall, we observed the expected differences in CSF neurodegeneration biomarker concentrations between the groups (results per cohort as Additional Material) (Additional file 1). Of interest, in the grey area group from the memory clinic cohort, the median T-tau and P-tau levels were slightly above the cut-off. T-tau levels were similar to the low Aß1-42 group but lower than the probable AD group (*p* < 0.001). P-Tau levels were higher in the grey area group compared to the low Aß1-42 group in both cohorts (*p* < 0.001 and *p* = 0.003, respectively).

### Neuropsychological profiles

The three groups differed in clinical severity and performance on several neuropsychological measures. The grey zone group was the least impaired, with lower clinical severity (median CDR score 0) and higher scores on global measures (median MMSE scores = 29 and MoCA scores = 24). However, the grey zone group did not differ from the low Aß_1-42_ group, which had a median CDR score corresponding to mild cognitive impairment (CDR 0.5) and lower scores on both the MMSE (median score 28) and MoCA (median score 22). The AD group showed significantly lower scores in assessments of clinical severity (median CDR = 0.5) and global cognitive status (median MMSE score = 23 and MoCA score = 18) than both the low Aß_1-42_ and grey zone groups (H(2) = 46.99, *p* < 0.05).

On neuropsychological measures, the grey zone group showed higher scores than the low Aß_1-42_ group on cognitive flexibility (TMT B), semantic verbal fluency, naming and some of the verbal memory tasks, specifically total learning and first trial in verbal serial learning. This pattern was even more pronounced when compared with the AD group, which nevertheless did not differ significantly from the low Aß_1-42_ group. The grey zone and low Aß_1-42_ groups showed similar performance in verbal delayed recall and recognition, with both groups displaying higher scores than the AD group.

**Fig. 2:**
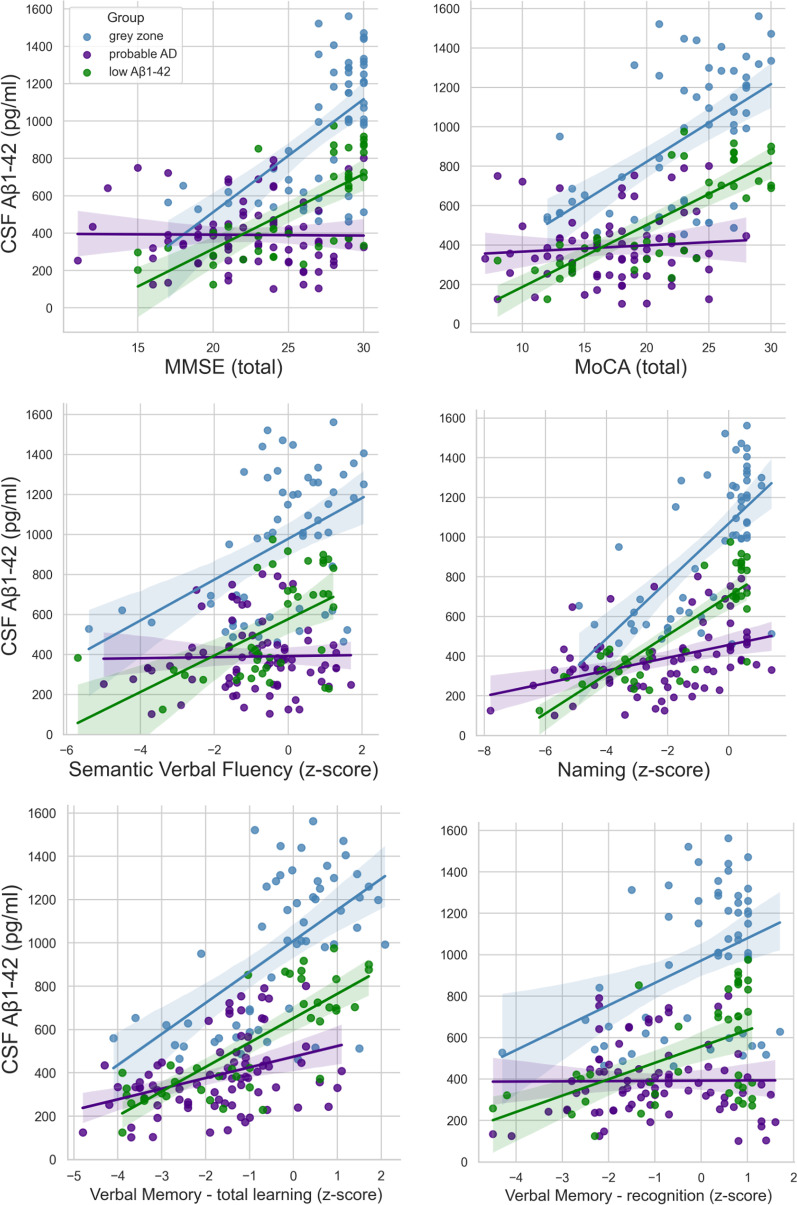
Association between Aß_1-42_ concentration (pg/ml) and neuropsychological performance per group (grey zone, probable AD, low Aß_1-42_). When comparing patients in the grey zone (blue) and those with low Aß_1-42_ (green) we observe an overlap both between the relative performance (z-scores in relation to performance of all participants) and patterns of association of Aß_1-42_ and cognitive performance. In contrast, patients with probable AD (purple) show lower scores in cognitive assessment

These overlapping patterns between groups are also reflected when looking at the association between Aß_1-42_ levels and neuropsychological performance per group (Table 2). Not only is there a similarity in the cognitive performance in the grey zone and low Aß_1-42_ groups, they also show similar patterns of a positive association between Aß_1-42_ levels and cognitive performance (Fig. 2). In contrast, patients in the AD group exhibited lower scores in cognitive screening tests and showed no association between Aß_1-42_ levels and neuropsychological performance.

**Table 2:** Association measures between Aß_1-__42_ concentration in CSF, neuropsychological performance and MRI visual ratings per group

	Aß_42_ concentration (pg/ml)								
	Grey Zone			Low Aβ_1-42_			AD		
	r_s_	95% CI		r_s_	95% CI		r_s_	95% CI	
		*LL*	*UL*		*LL*	*UL*		*LL*	*UL*
*Neuropsychological measures*									
MMSE (total)	0.53	0.31	0.70	0.69**	0.48	0.83	− 0.04	− 0.27	0.19
MoCA (total)	0.52	0.31	0.70	0.78**	0.61	0.88	0.09	− 0.16	0.32
Trail Making Test -Part A, z-score	0.18	− 0.12	0.46	0.53**	0.18	0.76	0.31*	0.01	0.56
Trail Making Test—Part B, z-score	− 0.69**	− 0.77	− 0.34	0.09	− 0.37	0.51	− 0.08	− 0.45	0.32
Semantic Verbal Fluency, z-score	0.40**	0.05	0.54	0.52**	0.24	0.72	− 0.01	− 0.24	0.22
Naming, z-score	0.64**	0.44	0.77	0.73**	0.53	0.85	0.43**	0.22	0.59
Verbal memory—total Learning, z-score	0.52**	0.29	0.69	0.74**	0.54	0.85	0.38**	0.17	0.56
Verbal memory—delayed recall, z-score	0.32**	0.05	0.55	0.44**	0.14	0.67	0.10	− 0.13	0.32
Verbal memory—intrusions, z-score	0.38**	0.12	0.59	0.37	0.05	0.62	0.40**	0.19	0.58
Verbal memory—recognition, z-score	0.22	− 0.05	0.47	0.33*	0.01	0.59	− 0.01	− 0.24	0.22
*MRI visual ratings*									
Medial temporal atrophy (MTA) score	− 0.20*	− 0.54	− 01	− 0.66**	− 0.81	− 0.42	− 0.05	− 0.28	0.19
Posterior atrophy (Koedam) score	− 0.02	− 0.30	− 0.26	− 0.21	− 0.35	− 0.31	0.16	− 0.09	0.38
WHM severity (Fazekas score)	− 0.30*	− 0.54	− 0.02	− 25	− 0.54	0.09	0.17	− 0.07	0.39

CI, confidence interval; *LL*, lower limit; *UL*, upper limit; MMSE, Mini-Mental State Examination; MoCA, Montreal Cognitive Assessment; WHM, White Matter Hyperintensities

*p < .05. **p < .001

### MRI visual ratings

The grey zone group showed less severe medial temporal atrophy than both the low Aß_1-42_ and AD groups. While around 40% of patients in the low Aß_1-42_ and AD groups showed severe MTA atrophy (MTA score equal to or greater than 2), the rates were much lower in the grey zone group, with only 7 (11.1%) and 11 (17.5%) patients showing severe MTA atrophy for the right and left hemispheres, respectively.

As shown in Table 3, the severity of posterior atrophy (Koedam score) was less severe in the grey zone group (17.5% of patients with a severe posterior atrophy score) than in the AD group (42.5%, *p* < 0.001), but similar to the low Aß_1-42_ group (28.6%, *p* = 0.204). The low Aß_1-42_ group and the AD group did not differ with respect to posterior atrophy.

**Table 3:** Neuropsychological and MRI characteristics of patients per group

Variable	Group				Test statistic and *p* value	*ES*
	N (n = 185)	Grey Zone (n = 63)	low Aß_1-42_ (n = 42)	AD (n = 80)		
*Neuropsychological measures, median (IQR)*						
MMSE, total score	174	29 (27–30)	28 (23–29)	23 (20–26)	H(2) = 46.987,* p* < 0.001	1.2
MoCA, total score	169	24 (21–27)	22 (16–27)	18 (14–21)	H(2) = 31.102, *p* < 0.001	0.9
TMT A (time to complete), z-score	119	− 0.43 (− 0.7–(− 0.2))	− 0.52 (− 0.8–(− 0.2))	− 0.2 (− 0.9–0.3)	H(2) = 2.989,* p* = 0.224	0.2
TMT B (time to complete), z-score	69	− 0.39 (− 0.6–0.1)	− 0.48 (− 0.7–(− 0.1))	0.34 (− 0.3–2.6)	H(2) = 10.586,* p* = 0.005	0.7
Semantic Verbal Fluency, z-score	175	− 0.01(− 0.8–0.8)	− 0.42(− 0.9–0.9)	− 0.75 (− 1.5–(− 0.1))	H(2) = 11.620,* p* = 0.003	0.5
Naming, z-score	173	0.12 (− 1.8–0.6)	− 1.25 (− 3.4–0.4)	− 1.9 (− 3.0–(− 0.4))	H(2) = 18.355, * p* < 0.001	0.6
Verbal memory—1^st^ trial learning, z-score	175	− 0.44 (− 1.0–0.4)	− 0.75 (− 2.8–0.1)	− 1.45 (− 2.8–(− 0.4))	H(2) = 16.904,* p* < 0.001	0.6
Verbal memory—total learning, z-score	175	− 0.07 (− 1.0–0.7)	− 1.0 (− 2.7–0.6)	− 1.45 (− 2.6–(− 0.8))	H(2) = 27.930,* p* < 0.001	0.8
Verbal memory—delayed recall, z-score	175	0.13 (− 0.9–0.9)	0.16 (− 1.3–0.8)	− 1.06 (− 1.8–0.8)	H(2) = 28.086,* p* < 0.001	0.8
Verbal memory—intrusions, z-score	172	− 0.69 (− 1.3–(− 0.4))	− 0.69 (− 2.3–− 0.4)	− 0.69 (− 3.4–0.1)	H(2) = 1.303,* p* = 0.521	0.1
Verbal memory—recognition, z-score	175	0.37 (− 0.7–0.9)	0.70 (− 1.2–0.8)	− 1.1 (− 2.1–0.2)	H(2) = 19.527,* p* < 0.001	0.7
*MRI visual ratings, median (IQR) or n* (%)						
MTA averaged score	160	0.85 (0–1)	1.51 (1–2)	1.25 (1–2)	H(2) = 19.8139,* p* < 0.001	0.7
Severe MTA—right hemisphere, n (%)	160	7 (11.1%)	17 (40.5%)	33 (41.2%)		
Severe MTA—left hemisphere, n (%)	160	11 (17.5%)	19 (45.2%)	35 (43.8%)		
Posterior atrophy (Koedam) score	161	1 (0–1)	1 (1–2)	2 (1–2)	H(2) = 13.484,* p* = 0.001	0.6
Severe posterior atrophy, n (%)	161	11 (17.5%)	12 (28.6%)	34 (42.5%)		
WMH severity (Fazekas score)	160	1 (0–1)	1 (1–2)	1(1–2)	H(2) = 5.922,* p* = 0.052	0.3
Severe WMH, n (%)	160	9 (14.3%)	13 (31%)	18 (22.5%)		

AD, Alzheimer’s disease; CDR, clinical dementia rating; ES, effect sizes; IQR, Interquartile range; MMSE, Mini-Mental State Examination; MoCA, Montreal Cognitive Assessment

The burden of white matter hyperintensities (Fazekas score) tended to be mild (median Fazekas score of 1) and was similar in all the groups. Severe WMH burden (Fazekas score above 2) was present in 9 (14.3%) patients in the grey zone group, 13 (31%) in the low Aß_1-42_ group and 18 (22.5%) in the AD group.

### Sensitivity analyses regarding role of P-Tau

To assess a possible modulation effect of P-Tau, we first compared patients within the grey zone group with respect to their P-Tau status (T- vs. T +). Corrected group comparisons show no differences between all clinical, neuropsychological and imaging variables, except for clinical severity. T + grey zone patients had a higher CDR score (Md 0.5) than T- grey zone patients (Md 0, IQR). Further corrected group comparisons (H(2) = 46.653, *p* < 0.001) also showed that the P-Tau/Aβ_1-42_ ratio was comparable (*p* = 0.370) between the low Aβ_1-__42_ (Md = 0.122, IQR = 0.017–0.172) and grey zone patients (Md = 0.024, IQR = 0.014–0.135), although it was lower in both groups than in the AD group (Md = 0.25, IQR = 0.066–0.353, both *p* < 0.001). We also found no significant association measures between P-Tau levels and clinical, neuropsychological and imaging variables per group and cohort (data known shown).

## Discussion

Our results suggest that patients in a CSF Aß_1-42_/Aß_1-40_ ratio grey zone, although less severely symptomatic, do show some overlap with patients on the AD continuum. Given the spectrum and dynamic processes associated with symptom presentation in AD, such overlap could translate into an increased risk of progressive cognitive impairment in a group of patients who would otherwise be classified as within the normal-range with respect to Aß- pathology when using the AT(N) dichotomic approach and therefore have a lower likelihood of AD.

Grey zone patients generally showed an unremarkable overall cognitive performance, showing a clear superior performance in cognitive measures than patients with a high likelihood of AD. This was predominantly found in executive functions and language tasks. But there were also some similarities, especially with the low Aß_1-42_ group. An overlap in performance between the grey zone and low Aß_1-42_ groups was found in global measures of clinical severity (CDR, MMSE, MoCA), but also in verbal episodic memory performance, specifically in delayed recall and recognition. This finding is of particular interest given the central role of markers of impaired consolidation, such as rapid forgetting and impaired recognition in memory tasks, in the typical cognitive presentation of AD. There is also evidence that memory decline appears to accompany Aβ accumulation, even in individuals within the Aβ-negative range [28]. The complex nature of the association between CSF biomarkers and clinical phenotypes over the disease course is also supported by the lack of an association between cognitive performance and Aß_1-42_ levels in the AD group, but not in the grey zone and low Aß_1-42_ groups.

The patterns of overlap and differences between all groups are somewhat mirrored by the MRI-based markers of brain structural integrity. Grey zone patients showed signs of a mild atrophy of the medial temporal lobe and posterior regions and mild WMH burden. In fact, less than 18% of participants in the grey zone group had severe medial temporal or posterior atrophy or severe WMH burden that could be interpreted as clinically relevant. In general, observed differences in structural brain changes could be partially explained by the fact that less severe atrophy is associated with early stages of the disease [9, 35, 40]. This seems to be the case for the grey zone patients, who showed a less severe clinical severity status and also scored higher in cognitive measures. Interestingly, however, the grey zone and Aß_1-42_ groups showed similar posterior atrophy and WMH burden scores, both of which are associated with a higher risk of clinical progression in AD [6, 13].

One of our aims was to explore a possible mediating role of P-Tau, as increases in CSF P-Tau may occur in response to early Aβ accumulation [30]. Our results suggest that the overlap in clinical characteristics between the groups does not appear to be mediated by the presence of abnormal P-Tau levels. This is also reinforced by comparable levels of P-tau/Aβ_1-42_ ratio, which is assumed to provide similar clinical information in the assessment of amyloid pathology [10], between the low Aβ_1-42_ and grey zone groups. The role of other possible mediators (e.g., ApoE genotype, inflammatory biomarkers) should be considered, but given the clinical nature of our data, these were not available for the current analyses.

The current results should be interpreted in the light of study-specific design and methodological characteristics. The comparison groups were chosen from a clinical perspective, with the AD group providing a sort of standard reference and the low Aβ_1-42_ depicting patients what would always be classified as A+ according to the AT(N), particularly in the absence of more specific information that could be provided from the Aβ ratio. Additionally, possible dissimilarities between these groups could also reflect differences, among others, in underlying pathophysiological processes, including CSF dynamics [10] and other Aβ associated pathologies [12].

The lack of guidelines on how best to operationalize non-dichotomous classifications of AD biomarkers contributes to inconsistencies between studies in the definition of peri-thresholds. Although we chose a less conservative approach that follows the data distribution of the cohort, in an effort to best reflect the specificities of such an unselected clinical cohort and assay-related factors, we obtained similar peri-threshold ranges as when using other previously proposed thresholds, such as the Erlangen score [34].

Some limitations of our study may have influenced the results. First, there are some a priori differences between the two cohorts. The ADNI cohort is a highly selected population recruited for research purposes, whereas our cohort represents an unselected clinical outpatient setting. Furthermore, there are differences in the assessment protocols, both for cognitive and biochemical measures, which we tried to minimize by the type of analyses performed. Secondly, the retrospective nature of the study is prone to selection bias. Finally, an important limitation is the lack of longitudinal data. Even in the ADNI cohort, as Aβ_1-40_ was only recently added to the study design, there were not enough data available to make longitudinal statistical comparisons with sufficient power.

Indeed, further information from longitudinal data to assess risk progression could better inform the clinical management of such borderline cases, particularly in terms of the length of follow-up required or the need for additional diagnostic procedures, as one hypothesis is that such patients may have been assessed at a very early stage of the disease, before amyloid positivity [38]. Thus, future studies should consider other sources of mediating effects (e.g., other types of biomarkers, genetic information), as well as other clinically relevant outcomes (e.g., neuropsychiatric symptoms or more sensitive measures of functional independence). Similarly, cross-validation in other types of cohorts would be required to extend such validation efforts across different assay methods and providers, especially considering the advantage of Aß_1-42_/Aβ_1-40_ as a more reliable biological marker for AD [16, 29].

## Conclusions

As other have suggested [38], dichotomous approaches do not reliably reflect the dynamic and longitudinal processes underlying the phenotypic manifestations of AD [17]. Our results contribute to the view that non- dichotomous approaches to Aß classification within the AT(N) system improve its reliability [8, 34, 38], namely by increasing its sensitivity. Even with the advent of blood-based biomarkers, diagnostic clarification by CSF analysis is still currently recommended to confirm plasma results [21, 23]. Thus, this study contributes to efforts to improve the clinical translation of CSF biomarkers within the AT(N) research framework and may guide clinical decisions in borderline cases. From the clinical overlap observed with patients on the AD continuum, it is conceivable that patients in the grey zone may be at an early stage of disease and at increased risk of progressive cognitive impairment, possibly associated with AD. Therefore, it is important to integrate clinical features into the AT(N) NIA-AA research framework for a more reliable translation into clinical practice. From a practical perspective, especially when other complementary diagnostic modalities are not available or not feasible (e.g., Aß- or Tau-PET, repeat CSF biomarkers), even for borderline cases with an inconspicuous cognitive and structural brain profile, clinical follow-up to better assess their risk of progression would be recommended.

## Supplementary Information

**Additional file 1**. **Additional Table 1.** Demographic and clinical characterization per group in the memory clinic cohort. **Additional Table 2.** Demographic and clinical characterization per group in the ADNI cohort.

## Data Availability

The datasets used and/or analysed during the current study are available either from ADNI or from the corresponding author on reasonable request, but subject to restrictions according to local law and ethic approvement.

## Abbreviations

Aß: Amyloid-beta
AD: Alzheimer’s disease
ADNI: Alzheimer's Disease Neuroimaging Initiative
CERAD-NAB: CERAD Neuropsychological Assessment Battery
CDR: Dementia Rating Scale
CSF: Cerebrospinal fluid
MMSE: Mini-Mental State Examination
MoCA: Montreal Cognitive Assessment
MRI: Magnetic Resonance Imaging
MTA: Medial temporal atrophy
NIA-AA: National Institute on Aging and Alzheimer’s Association
PET: Positron-emission tomography
TMT: Trail Making Test
WMH: White matter hyperintensities
