# Supplementary material for: Clinical characteristics of patients with suspected Alzheimer’s disease within a CSF Aß-ratio grey zone
Source: Neurol Res Pract. 2023 Aug 3;5:40. doi: 10.1186/s42466-023-00262-8 (PMC10398972; doi:10.1186/s42466-023-00262-8)
Supplement: Supplementary file 1 — Additional file 1. Additional Table 1. Demographic and clinical characterization per group in the memory clinic cohort. Additional Table 2. Demographic and clinical characterization per group in the ADNI cohort. [file 42466_2023_262_MOESM1_ESM.docx]

Additional Table 1. Demographic and clinical characterization per group in the memory clinic cohort

|  | **Group** | | |  |
| --- | --- | --- | --- | --- |
| **Variable** | **Grey zone**  **(n=31)** | **low Aß_42_**  **(n=23)** | **AD**  **(n=50)** | ***p*-value** |
| **Demographics** | | | | |
| Age at visit in years, median (IQR) | 72 (69-76) | 74 (65-81) | 72 (68-77) | 0.757 |
| Female sex, n (%) | 21 (67.7%) | 9 (39.1%) | 31(62%) | 0.086 |
| Education in years, median (IQR) | 11 (9-12) | 12 (11-14) | 11 (9-13) | 0.103 |
| Dementia in first degree relatives, n (%) | 5 (16.1%) | 10 (43.4%) | 19 (38%) | 0.2 |
| **Previous medical history, n (%)** | | | | |
| Arterial hypertension | 17 (54.8%) | 13 (56.5%) | 34 (68%) | 0.424 |
| Diabetes mellitus | 6 (19.4%) | 3 (13%) | 5 (10%) | 0.288 |
| Dyslipidemia | 15 (48.4%) | 13 (56.5%) | 24 (48%) | 0.777 |
| OSAS | 1 (3.2%) | 0 (0%) | 2 (4%) | 0.63 |
| Smoking | 6 (19.4%) | 6 (26%) | 11 (22%) | 0.977 |
| Depression | 5 (16.1%) | 4 (17.4%) | 6 (12%) | 0.789 |
| Ischemic stroke/TIA | 1 (3.2%) | 5 (21.7%) | 6 (12%) | 0.333 |
| Intracerebral hemorrhage | 2 (6.5%) | 1 (4.3%) | 1 (2%) | 0.593 |
| Coronary heart disease | 3 (9.7%) | 3 (13%) | 5 (10%) | 0.908 |
| Myocardial infarction | 2 (6.5%) | 1 (4.3%) | 2 (4%) | 0.876 |
| Atrial fibrillation | 0 (0%) | 4 (17.4%) | 7 (14%) | 0.067 |
| **Clinical severity** |  |  |  | 0.685 |
| CDR score, median (IQR) | 0.5 (0.5-0.5) | 1 (0.5-1) | 0.5 (0.5-1) | 0.057 |
| **CSF biomarkers, median (IQR)** | | | | |
| Aß_1-42_ (pg/mL) | 560 (517-686) | 322 (272-399) | 316 (240-357) | <0.001 |
| Aß_1-40_ (pg/mL) | 10601 (9869-12724) | 5989 (4716-7637) | 9603 (7543-11640) | <0.001 |
| Aß_1-42_/Aß_1-40_ ratio | 0.52 (0.50-0.55) | 0.52 (0.52-0.56) | 0.32 (0.25-0.37) | <0.001 |
| T-Tau (pg/mL) | 485 (397-699) | 359 (254-465) | 676 (552-1041) | <0.001 |
| P-Tau (pg/mL) | 79 (65-106) | 57 (46-69) | 95 (80-125) | <0.001 |

Abbreviations: AD=Alzheimer’s disease, CDR= Clinical Dementia Rating scale, CSF= Cerebrospinal fluid, IQR= Interquartile range, MCI=mild cognitive impairment, OSAS=obstructive sleep apnea syndrome, TIA=transient ischemic attack

Additional Table 2. Demographic and clinical characterization per group in the ADNI cohort

|  | **Group** | | |  |
| --- | --- | --- | --- | --- |
| **Variable** | **Grey Zone**  n=32 | **low Aß_1-42_**  n=19 | **AD**  n=30 | **P- value** |
| **Demographics** | | | | |
| Age at visit in years, median (IQR) | 71 (67-75) | 72 (67-77) | 72 (66-78) | 0.851 |
| Female sex, n (%) | 19 (59%) | 10 (52.6%) | 10 (33.3%) | 0.120 |
| Education in years, median (IQR) | 18 (16-19) | 18 (16-19) | 16 (14-19) | 0.57 |
| Dementia in first degree relatives, n (%) | 16 (50%) | 11 (58%) | 12 (40%) | 0.578 |
| **Previous medical history, n (%)** | | | | |
| Arterial hypertension | 10 (31.3%) | 9 (47.4%) | 12 (40%) | 0.373 |
| Diabetes mellitus | 4 (12.5%) | 1 (5.3%) | 2 (6.7%) | 0.710 |
| Dyslipidemia | 17 (53.1%) | 9 (47.4%) | 14 (46.6%) |  |
| OSAS | 3 (9.4%) | 4 (21%) | 2 (6.7%) | 0.435 |
| Smoking | 2 (6.3%) | 4 (21%) | 2 (6.7%) | 0.361 |
| Depression | 8 (25%) | 2 (10.5%) | 12 (40%) | 0.443 |
| Ischemic stroke/TIA | 1 (3.1%) | 0 (0%) | 0 (0%) |  |
| Coronary heart disease | 1 (3.1%) | 1 (5.5%) | 1 (3.3%) |  |
| **Clinical severity** | | | | |
| CDR score, median (IQR) | 0 (0-0) | 0 (0-0.5) | 1 (0.5-1) | <0.001 |
| **CSF biomarkers**, **median (IQR)** | | | | |
| Aß_1-42_ (pg/mL) | 1211 (1074-1327) | 833 (699-872) | 518 (430-639) | <0.001 |
| Aß_1-40_ (pg/mL) | 18815 (16655-21265) | 12390 (11140-14660) | 16820 (12880-18880) | <0.001 |
| Aß_1-42_/Aß_1-40_ ratio | 0.64 (0.60-0.69) | 0.62 (0.59-0.64) | 0.34 (0.29-0.41) | <0.001 |
| T-Tau (pg/mL) | 214 (184-259) | 154 (134-181) | 334 (265-430) | <0.001 |
| P-Tau (pg/mL) | 18 (16-25) | 13 (11-15) | 33 (25-40) | <0.001 |

Abbreviations: AD=Alzheimer’s disease, CDR= Clinical Dementia Rating scale, CN=cognitive normal, CSF= Cerebrospinal fluid, IQR= Interquartile range, MCI=mild cognitive impairment, OSAS=obstructive sleep apnea syndrome, TIA=transient ischemic attack
